# Editorial: From Structure to Function - The Interplay Between Cell Adhesion Molecules and the Cytoskeleton

**DOI:** 10.3389/fcell.2020.00104

**Published:** 2020-02-25

**Authors:** Vladimir Sytnyk, Thomas Fath

**Affiliations:** ^1^School of Biotechnology and Biomolecular Sciences, The University of New South Wales, Sydney, NSW, Australia; ^2^Department of Biomedical Sciences and Dementia Research Centre, Faculty of Medicine and Health Sciences, Macquarie University, Sydney, NSW, Australia

**Keywords:** cell adhesion molecule, neuronal synapse, immunological synapse, cytoskeleton, endothelial cell, intracellular signal transduction, fasciculation

There are fundamental similarities in how different cells interact with and organize functional contacts with other cells, and how these interactions result in the structural changes in cells and tissues ultimately leading to highly defined functions. The aim of this Research Topic is to highlight some of the key players at the intersection of cell adhesion molecule-mediated cell-cell interaction and changes in the cell architecture regulating eukaryotic cell function.

In the developing nervous system, cell-to-cell interactions play a key role in navigating growing axons to the correct targets. Cell adhesion molecules mediating these interactions are expressed in the highly regulated spatial and temporal manner determined by developmental programs, which remain poorly understood, but play key roles in the establishment of neuronal circuitries. Mohan et al. demonstrates that the expression of NrCAM in the amygdalar pathway during development is necessary for fasciculation of the stria terminalis (ST) nerve fiber bundle interconnecting the central amygdala and bed nucleus of the ST. The functional importance of the ST fasciculation is underscored by impairments in contextual fear conditioning in NrCAM null mice, which the authors report.

NrCAM belongs to the L1 family of cell adhesion molecules of the immunoglobulin superfamily. Its members accumulate in neuronal synapses in the mature nervous system, where they regulate synaptic efficacy and maintain the stability of synaptic contacts (Sytnyk et al., 2017). At synapses, L1 family members and other cell adhesion molecules, such as the neural cell adhesion molecule (NCAM), are linked with the subcortical cytoskeleton by adaptor proteins including ankyrins (Leshchynsḱa and Sytnyk, 2016). Using Drosophila as a model organism, Weber et al. revealed a new aspect of these interactions by interrogating the interdependency of the synaptic localization of fasciclin II (NCAM homolog) and neuroglian (L1 homolog) and different ankyrin isoforms at neuromuscular synapses. Their study provides a hierarchical model in which the presynaptic localization of giant ankyrin isoform Ank2-L is required for the proper localization of Ank2-XL, fasciclin II, and neuroglian. Ank2-XL in turn is critical for proper synaptic microtubule organization. These findings advance our knowledge on the regulation of synaptic adhesion molecules and ankyrins, which was previously characterized at the neuromuscular junction (Pielage et al., 2008) and at the glutamatergic synapse in the rat brain (Smith et al., 2014).

The neuronal synapse, although plastic in nature, is a long-lasting cell-cell contact, whereas the immunological synapse is usually a transient interaction between T cells and antigen-presenting cells. However, the interactions between these cells also involve a very precise program of the interactions between cell adhesion molecules and the cytoskeleton culminating in the formation of a functional immunological synapse. Two contributions to this Research Topic discuss the axis of cell adhesion molecule-mediated signaling to the cytoskeleton in these cells. Firstly, a perspective Roy and Burkhardt, introduces the integrin-dependent sub-cellular organization of the actin cytoskeleton in T cells, which is followed by Martín-Cófreces et al. which provides an in-depth discussion on the signaling mechanisms that are involved in the communication between cell adhesion molecules and the cytoskeleton.

While the contacts between endothelial cells are not typically called synapses, their formation and regulation also depend on the cell-to-cell recognition and structural reorganization of the cells mediated by the cell adhesion molecules and cytoskeletal proteins, respectively. The regulation of the cell adhesion molecules and cytoskeletal proteins at desmosomes, adherens junctions and tight junctions between epithelial cells by the protein phosphatase 2A is reviewed in the contribution Schuhmacher et al. In particular, a critical role of Protein phosphatase 2A in stabilizing the adhesion complexes, such as the E-cadherin/β-catenin adhesion complex is discussed.

This Research Topic highlights fundamental similarities in the molecular and developmental programs leading to the contact formation, maintenance and remodeling between different types of cells, which substantially differ in their functions. However, many questions remain unanswered. For example, the role of cell adhesion molecules and actin cytoskeleton as mechanosensors and mechanotransducers in immunological synapses is well-established as discussed by Roy and Burhardt while mechanoregulation of neuronal synapses remains poorly understood (Kilinc, 2018). Understanding of the similarities between different cell-cell contacts may help in development of the novel or repurposing of the existing therapeutics targeting cell adhesion and the cytoskeleton in various disorders associated with abnormal cytoarchitecture.

## Author Contributions

All authors listed have made a substantial, direct and intellectual contribution to the work, and approved it for publication.

### Conflict of Interest

The authors declare that the research was conducted in the absence of any commercial or financial relationships that could be construed as a potential conflict of interest.
